# ASO Author Reflections: Enhancing Shared Decision-Making with Risk Prediction Models in Sarcoma Care

**DOI:** 10.1245/s10434-025-16970-1

**Published:** 2025-01-29

**Authors:** Leti van Bodegom-Vos, Anouk Kruiswijk, Michiel van de Sande

**Affiliations:** 1 Department of Biomedical Data Sciences, Medical Decision Making, Leiden University Medical Center, Leiden, The Netherlands; 2 Orthopedic Surgery, Leiden University Medical Center, Leiden, The Netherlands

## Past

Risk prediction models (RPMs) have become essential tools in modern oncology, supporting clinicians in making data-driven, personalized treatment decisions. These statistical tools predict individual patient outcomes on the basis of clinical characteristics, enhancing evidence-based medicine and shared decision-making. RPMs have found applications across various conditions, including breast cancer and currently in soft tissue sarcoma (STS) management. Tools such as Sarculator^1^ and PERSARC,^2^ designed specifically for STS, estimate risks of recurrence and survival using clinical parameters to tailor sarcoma treatment strategies.

Despite their external validation in several international sarcoma patient cohorts and their potential to support decision-making, the integration of RPMs into routine sarcoma care remains limited and poorly understood.^3^ This study evaluated the use of RPMs in multidisciplinary tumor boards and patient consultations worldwide, and the factors that influence their adoption.^4^ It also explored clinicians’ perspectives on shared decision-making in STS treatment and how RPMs can support this process. By addressing the barriers of integrating RPMs into clinical workflows, the study aimed to enhance collaborative decision-making and advance personalized sarcoma care.

## Present

Our study provides valuable insights into the use of risk prediction models (RPMs) in soft tissue sarcoma (STS) patient care and their role in shared decision-making (SDM). Among 278 surveyed clinicians 68% reported using RPMs in multidisciplinary tumor boards, with 27% using them frequently or consistently. Sarculator and PERSARC were the most frequently used tools, valued for evaluating (neo)adjuvant chemotherapy benefits and providing prognostic insights. However, concerns about the reliability of predictions and a lack of guidelines were significant barriers to wider adoption. During patient consultations, 65% of respondents reported using RPMs, though only 20% used them frequently or consistently. In the consultation room, RPMs were primarily used for prognostic estimates and treatment planning. However, their use was limited by concerns about patient anxiety and applicability to individual cases.

Clinicians noted that approximately half of patients with STS face multiple viable treatment options, highlighting the need for personalized discussions. Despite challenges, clinicians showed strong motivation for SDM, reflected in a high median incorpoRATE score of 83.8 out of 100.

These findings highlight a critical gap: while RPMs have significant potential to support treatment decisions and clinicians recognize the need for personalized decision-making—given that many patients encounter multiple viable options—the integration of RPMs into clinical workflows, particularly during patient consultations, remains incomplete.

## Future

To effectively integrate RPMs into clinical workflows and enhance collaborative decision-making in sarcoma care, future implementation efforts should focus on the following:

1. Establishing clear guidelines for RPM use. International and national guidelines should provide standardized recommendations on when and how to use RPMs to ensure consistent application across diverse clinical settings. The European Society for Medical Oncology (ESMO) recently emphasized the importance of risk stratification for sarcoma treatment decisions, including whether chemotherapy should be considered.^5^ However, while ESMO suggests incorporating clinical and pathological factors alongside clinical judgment, it does not mandate the use of RPMs.
2. Building trust and understanding in RPMs. Clinicians need training to use RPMs effectively, interpret outcomes accurately, and apply calculated risks to individual patients. Validating RPMs on local cohorts and routinely updating these models can further enhance trust by ensuring reliability in specific contexts.
3. Improving communication skills and patient engagement. Effective communication training is essential for clinicians to present RPM-derived prognostic estimates in ways that reduce patient anxiety and support meaningful shared decision-making. Such training should emphasize clear, empathetic, and patient-centered discussions, actively engaging patients in decisions that align with their preferences, values, and treatment goals. Recognizing that maximum survival may not always be a patient’s primary objective,^6^ clinicians must navigate multiple treatment options tailored to individual patient and tumor characteristics.

In addition to improving the integration of RPMs in sarcoma care, it is crucial to prevent similar gaps from arising with newly developed RPMs in other areas of oncology. To achieve this, we recommend that future research on RPM effectiveness in oncology adopt hybrid implementation-effectiveness study designs. These studies assess both clinical effectiveness and implementation within a single framework, providing practical insights for real-world application. By involving stakeholders early and addressing implementation challenges from the start, hybrid designs help ensure a smoother transition from research to practice. Moreover, by incorporating factors such as patient preferences, clinician practices, and contextual constraints, these studies enhance generalizability and can accelerate the adoption of RPMs across oncology.
